# GSK3 inhibitor enhances gemtuzumab ozogamicin‐induced apoptosis in primary human leukemia cells by overcoming multiple mechanisms of resistance

**DOI:** 10.1002/jha2.600

**Published:** 2022-12-12

**Authors:** Aki Inase, Yimamu Maimaitili, Shiro Kimbara, Yu Mizutani, Yoshiharu Miyata, Shinya Ohata, Hisayuki Matsumoto, Akihito Kitao, Rina Sakai, Koji Kawaguchi, Ako Higashime, Shigeki Nagao, Keiji Kurata, Hideaki Goto, Shinichiro Kawamoto, Kimikazu Yakushijin, Hironobu Minami, Hiroshi Matsuoka

**Affiliations:** ^1^ Division of Bioresource Research and Development Department of Social/Community Medicine and Health Science Kobe University Graduate School of Medicine Kobe Japan; ^2^ Division of Medical Oncology and Hematology Department of Medicine Kobe University Graduate School of Medicine Kobe Japan; ^3^ Department of Clinical Laboratory Kobe University Hospital Kobe Japan; ^4^ Department of Medical Oncology/Hematology Konan Medical Center Kobe Japan; ^5^ Department of Hematology and Oncology Kita‐harima Medical Center Ono Japan; ^6^ Department of Transfusion and Cell Therapy Kobe University Hospital Kobe Japan; ^7^ Cancer Center, Kobe University Hospital Kobe Japan

**Keywords:** AML, gemtuzumab ozogamicin, GSK3 inhibitor, overcome resistance

## Abstract

In acute myeloid leukemia (AML), the heterogeneity of genetic and epigenetic characteristics makes treatment difficult. The prognosis for AML is therefore poor, and there is an urgent need for new treatments for this condition. Gemtuzumab ozogamicin (GO), the first antibody‐drug conjugate (ADC), targets the CD33 antigen expressed in over 90% of AML cases. GO therefore has the potential to counter the heterogeneity of AML patients. However, a major clinical problem is that drug resistance to GO diminishes its effect over time. Here, we report that the inhibition of glycogen synthase kinase 3 (GSK3) alone overcomes several forms of GO resistance at concentrations without antileukemic effects. The GSK3 inhibitors tested significantly enhanced the cytotoxic effect of GO in AML cell lines. We elucidated four mechanisms of enhancement: (1) increased expression of CD33, the target antigen of GO; (2) activation of a lysosomal function essential for hydrolysis of the GO linker; (3) reduced expression of MDR1 that eliminates calicheamicin, the payload of GO; and (4) reduced expression of the anti‐apoptotic factor Bcl‐2. A similar combination effect was observed against patient‐derived primary AML cells. Combining GO with GSK3 inhibitors may be efficacious in treating heterogeneous AML.

## 1 INTRODUCTION

Acute myeloid leukemia (AML) is the most common leukemia and has a high incidence in the elderly (median 68 years) [1, 2]. Induction chemotherapy results in complete remission in 40%–60% of patients aged over 60, but 50% of these patients experience recurrence and their subsequent prognosis is poor [3]. One factor in these outcomes is the heterogeneity of AML, with various gene mutations and epigenetic changes. Various molecular‐targeted therapies aimed at improving the effects of therapy have been developed, such as isocitrate dehydrogenase inhibitors and FLT3 inhibitors, and have been shown to be effective against aggressive AML. Unfortunately, however, these can only be applied to patients with specific mutations, and resistance is quickly acquired [4]. Antibody‐drug conjugates (ADCs) are composed of three major components: monoclonal antibodies, linkers, and cytotoxic payloads. ADCs can deliver highly cytotoxic payloads directly to tumor cells while sparing normal cells and can be used to achieve high lethality against targeted cancer cells. Gemtuzumab ozogamicin (GO) is the only ADC specific for AML. GO binds to CD33‐expressing leukemia cells through its humanized anti‐CD33 monoclonal antibody portion and then kills them with its calicheamicin portion [5, 6, 7, 8, 9]. Since CD33‐expressing leukemia cells are commonly found in leukemia cells having various gene mutations, this effect was expected. The Food and Drug Administration approved GO in 2017 as a single agent for the treatment of newly diagnosed CD33‐positive AML and relapsed or refractory CD33‐positive AML [10, 11, 12]. In 2018, the European Medicines Agency also approved GO for concomitant use only for newly diagnosed CD33‐positive AML [13]. Unfortunately, the remission rate for relapsed/refractory AML after administration of GO is ∼30% [14, 15, 16].

GO does not show efficacy in some patients with AML, and resistance is attributed to various mechanisms. The efficacy of GO is attenuated by differences in the expression of CD33 levels, decreased lysosomal function, excretion of calicheamicin prior to nuclear transport by expression of multidrug resistance factors, and expression of anti‐apoptotic factors [17, 18]. To fully exert its effects, GO must first be transported into cells after binding to cell surface CD33. The linker site of internalized GO then must be degraded by lysosomes to release calicheamicin, which migrates to the nucleus to bind to DNA and cause single‐strand or double‐strand breaks, causing cell death. Rapid excretion of intracellular calicheamicin and expression of antiapoptotic factors can thus severely attenuate the cell death‐inducing effect of calicheamicin.

In previous studies, in AML cell lines and primary human AML cells, we succeeded in enhancing the effect of GO by using mTORC1/2 inhibitors as lysosomal function activators [19, 20]. However, simply activating lysosomal function did not enhance the effect of GO in all AML cell types. Therefore, we screened for more effective inhibitors. To do so, we focused on the PI3K/AKT/mTOR signaling pathway and found that glycogen synthase kinase 3 (GSK3) inhibitors can successfully overcome resistance to GO.

## 2 MATERIALS AND METHODS

### 2.1 Cell lines and cell culture

Human AML cell lines human promyelocyte leukemia cell line (HL‐60) and KO52 (JCRB Cell Bank), U937 American Type Culture Collection (ATCC), human monocytic leukemia cell line (THP‐1) (ECACC), MARIMO (kind gift from Dr. Yosuke Minami at the Department of Hematology/Oncology, Kobe University, Kobe, Japan), and NB4 (kind gift from Dr. Hideki Nakakuma at the Department of Hematology/Oncology, Wakayama Medical University, Wakayama, Japan) were cultured as previously described [19, 20]. The feeder cell line Hs27 was obtained from ATCC. All cell lines were cultured as previously described [19, 20]. To authenticate cell lines, short tandem repeat analysis was performed by BEX using GenePrint 10 System (Promega). Mycoplasma testing was carried out routinely using an e‐Myco Plus Mycoplasma polymerase chain reaction (PCR) Detection Kit (iNtRON Biotechnology), and all cell lines were negative for mycoplasma.

### 2.2 Reagents and antibodies

Chemical reagents and antibodies used in this study are listed in Table S1.

### 2.3 Culture of primary human leukemia cells

All bone marrow aspirates were taken from routine diagnostic specimens after the written informed consent of patients with AML or myelodysplastic syndrome (MDS). The project received approval from the ethics committees of Kobe University (#170216, #180288) and was conducted in accordance with the Helsinki Declaration. Mononuclear cells were isolated from bone marrow samples by Ficoll with SepMate (STEMCELL Technologies), according to the manufacturer's instructions, and cryopreserved by Cell Banker until use. The human leukemia cells were cultured in accordance with protocols published elsewhere [21, 22].

### 2.4 Apoptosis assay

**FIGURE 1:**
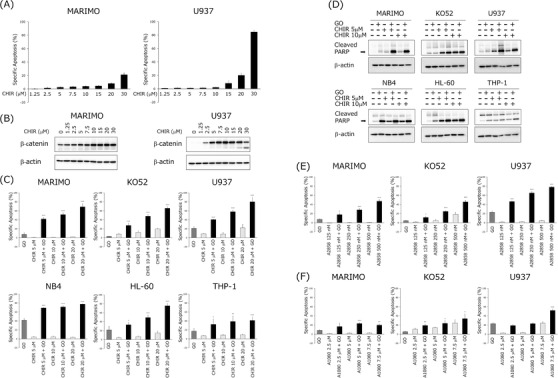
Cytotoxic effect of gemtuzumab ozogamicin (GO) was enhanced by GSK3α/β inhibitors treatment in acute myeloid leukemia (AML) cell lines. For all experiments, specific apoptosis was determined after 48 h of incubation. The resulting data were expressed as means ± standard deviation (SD) of three independent experiments. (A) U937 and MARIMO cells were treated with the indicated concentrations of CHIR99021 (CHIR). Apoptosis was determined by staining with Annexin V and Propidium Iodide (PI), followed by flow cytometry. (B) U937 and MARIMO cells were treated with the indicated concentrations of CHIR and then analyzed for accumulation of β‐catenin by western blotting. (C) Cells were treated with 2.5 μg/ml, 0.5 μg/ml (for THP‐1), or 0.25 μg/ml (for NB4) of GO alone, or in combination with CHIR. *, **, and *** indicate *p* < 0.05, *p* < 0.01, and *p* < 0.001, respectively. (D) Cells were treated with GO, CHIR, or GO+CHIR. Whole cell lysates were analyzed by western blotting for poly(ADP‐ribose) polymerase (PARP). β‐actin was used as a loading control. (E) MARIMO, KO52, and U937 cells were treated with GO, AZD2858 (A2848), or GO+A2858. The statistical significance of differences observed between GO and GO+A2858 were determined using Student's *t*‐test. *and *** indicate *p* < 0.05 and *p* < 0.001, respectively. (F) MARIMO, KO52, and U937 cells were treated with GO, AZD1080 (A1080), or GO+A1080.The statistical significance of differences observed between GO and GO+A1080 was determined using Student's *t*‐test. *, ** and *** indicate *p* < 0.05, *p* < 0.01, and *p* < 0.001, respectively.

Apoptosis assays were performed as previously described [19, 20]. AML cells were seeded in growth medium at a concentration of 2.5 × 10^5^ cells/ml. Human AML cells were seeded at the same density on Mitomycin C‐treated Hs27 feeder cells in IMDM medium (containing various cytokines). Cells were exposed to GO and GSK3α/β inhibitors individually, or in combination, for 48 h. Concentrations of GO were 0.25 μg/ml for NB4, 0.5 μg/ml for THP‐1, and 2.5 μg/ml for other cells. GSK3α/β inhibitors were treated at low concentrations to prevent single‐agent cell death. The treatment concentrations are shown in Figure 1. After 48 h, cells were stained with the Annexin V‐FITC Apoptosis Detection Kit (Nacalai Tesque) and analyzed using a BD FACSVerse flow cytometer (BD Biosciences). The percent specific apoptosis = stimulation‐induced apoptosis – spontaneous apoptosis. The combination index (CI) was calculated as: CI = (EA + EB)/EAB, where EA and EB represent the effect of the individual drugs (percentage of dead cells), and EAB represents the effect (percentage of dead cells) that results from the combination of the two drugs. CI values ​​were evaluated as additive (CI = 1), synergistic (CI < 1), and antagonistic (CI > 1) [19, 23].

### 2.5 Analysis of CD33 expression

AML cells were seeded in a growth medium at 2.5 × 10^5^ cells/ml and exposed to CHIR99021 at concentrations of 0, 5, 10, 20 μM for 48 h. After harvesting the cells, they were labeled with antihuman CD33‐FITC (eBioscience) or Mouse IgG1 κ‐FITC (eBioscience). After incubation at 4°C for 30 min, cells were analyzed on a BD FACSVerse flow cytometer.

### 2.6 Assessment of MDR‐1 activity

Rh123 functions as an excellent substrate for MDR‐1, and the MDR‐1 inhibitor verapamil has been shown to enhance the retention of Rh123 in MDR‐1‐expressing cells. To measure Rh123 uptake, the KO52 cell line was seeded at 2.5 × 10^5^ cells/ml and treated with 5 μM CHIR99021 for 24 or 48 h and verapamil at concentrations of 10, 20, 30, 40, and 50 μM for 48 h. After incubation, 200 ng/μl Rh123 was added, and the incubation was continued at 37°C for 1 h. Cells were then collected, washed with phosphate buffered saline, replenished with fresh medium, then incubated at 37°C for 1 h. After harvesting, the cells were stained with 7‐AAD and analyzed with a BD FACSVerse flow cytometer.

### 2.7 Quantitative real‐time PCR

Quantitative evaluation of ABCB1 or Bcl‐2 expression was performed using the OneStep TB Green PrimeScript PLUS reverse transcription PCR (RT‐PCR) Kit (Perfect Real Time) (Takara). Glyceraldehyde‐3‐phosphate dehydrogenase (GAPDH) was used as a control. Relative expression levels were calculated by the Comparative Cycle Threshold (Ct) method. All PCRs were performed three times on the Thermal Cycler Dice RealTime System (Takara). All primer sequences are listed in Table S2.

## 3 RESULTS

### 3.1 GSK3α/β inhibitors synergistically enhance GO‐induced toxicity in AML cells

To investigate the effect of GSK inhibition on GO efficacy, we determined the concentrations at which the GSK3α/β inhibitor CHIR99021 caused less than 20% apoptosis when added to AML cells. No cytotoxicity was observed in MARIMO cells with CHIR99021 concentrations up to 30 μM, but strong toxicity was observed in U937 cells at that concentration (Figure 1A). The accumulation of β‐catenin promoted by GSK3α/β inhibition was observed in both cell lines at concentrations of CHIR99021 above 5 μM (Figure 1B). These results indicate that CHIR99021 inhibits GSK3α/β activity in the concentration range of 5–20 μM, with minimal cytotoxic effects.

Next, the effect of CHIR99021 on the cytotoxicity of GO in 6 human AML cell lines (MARIMO, KO52, U937, NB4, HL‐60, and THP‐1) was investigated. In all cell lines, the combination of GO and CHIR99021 significantly enhanced apoptosis compared to GO (2.5 μg/ml) alone or CHIR99021 (5–20 μM) alone (Figure 1C). The CI of all cell lines was <1 at all concentrations of CHIR99021, indicating that CHIR99021 synergistically amplified the toxicity of GO (Figure S1A). The addition of CHIR99021 to GO enhanced the expression of the apoptosis‐dependent marker‐cleaved PARP in all cell lines used compared to that of GO alone (Figure 1D). Three cell lines, MARIMO, KO52, and U937, were used to investigate the effects of the GSK3α/β inhibitors AZD2858 and AZD1080. We first determined the concentration‐dependence of the toxicity of the inhibitors (Figures S1B,C). Each enhanced toxicity when combined with GO. A particularly high combinatorial effect was observed with AZD2858 (Figure 1E,F). The results show that combined treatment with GO and GSK3α/β inhibitors enhanced apoptosis in leukemia cell lines.

### 3.2 GSK3α/β inhibitor increases CD33 expression on AML cell lines

**FIGURE 2:**
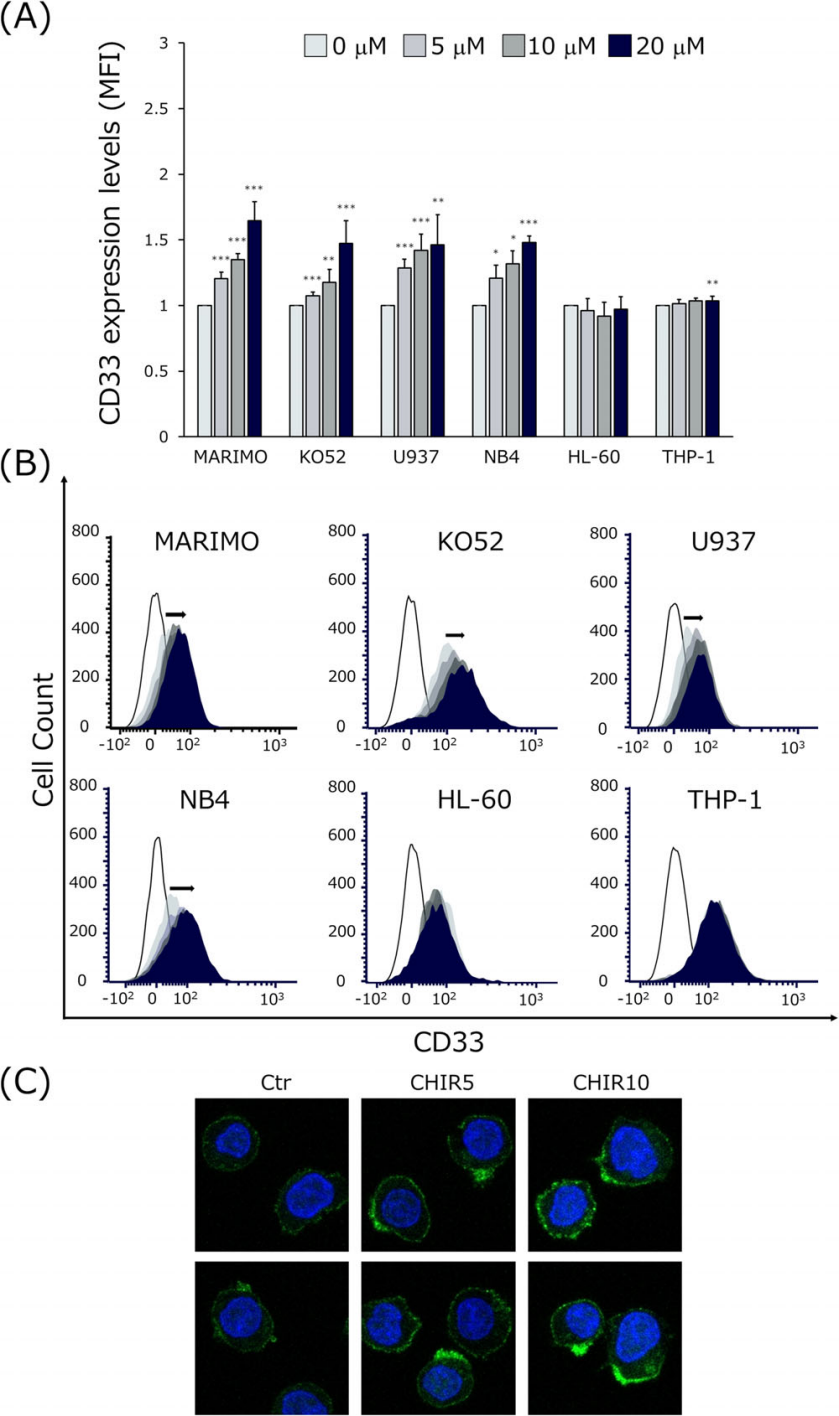
GSK3α/β inhibitor increases CD33 expression on acute myeloid leukemia (AML) cell lines. (A) Flow cytometric analysis of CD33 expression after 48 h at different concentrations of CHIR99021. (B) The bar graph shows CD33 expression at different drug concentrations. The heights of the bars are CD33 mean fluorescence intensity (MFI) from CHIR‐treated cells normalized to CD33 MFI of untreated cells. Data are expressed as means ± SD of three independent experiments. *, ** and *** indicate *p* < 0.05, *p* < 0.01, and *p* < 0.001, respectively. (C) U937 cells were treated with CHIR 5 μM and analyzed CD33 expression by confocal microscopy.

To investigate the mechanism of enhancement of GO toxicity by GSK3α/β inhibitors, we first examined the expression level of CD33, which is important for the intracellular internalization of GO. The expression level of CD33 is positively correlated with the effects of GO [24, 25]. Previous studies showed the expression level of CD33 in MARIMO cells was lower than in other cell lines, which was thought to explain why GO‐induced cell death was significantly reduced in them [19, 20]. CD33 expression increased in a concentration‐dependent manner 48 h after CHIR99021 treatment in the cell lines MARIMO, KO52, U937, and NB4. In contrast, almost no changes were observed in HL‐60 and THP‐1 cells (Figure 2A,B). To confirm these data, surface levels of CD33 in U937 cells were analyzed by immunofluorescence. Consistent with the results of flow cytometry, the expression of CD33 was enhanced in a CHIR99021 concentration‐dependent manner (Figure 2C). Thus, GSK3α/β inhibitors may promote internalization of GO by enhancing the expression of CD33, to which the antibody portion of GO binds, thus enhancing cytotoxicity (even in cell lines with low CD33 expression, such as MARIMO cells [19]).

### 3.3 GSK3α/β regulates lysosome biogenesis in an mTORC1‐dependent manner

**FIGURE 3:**
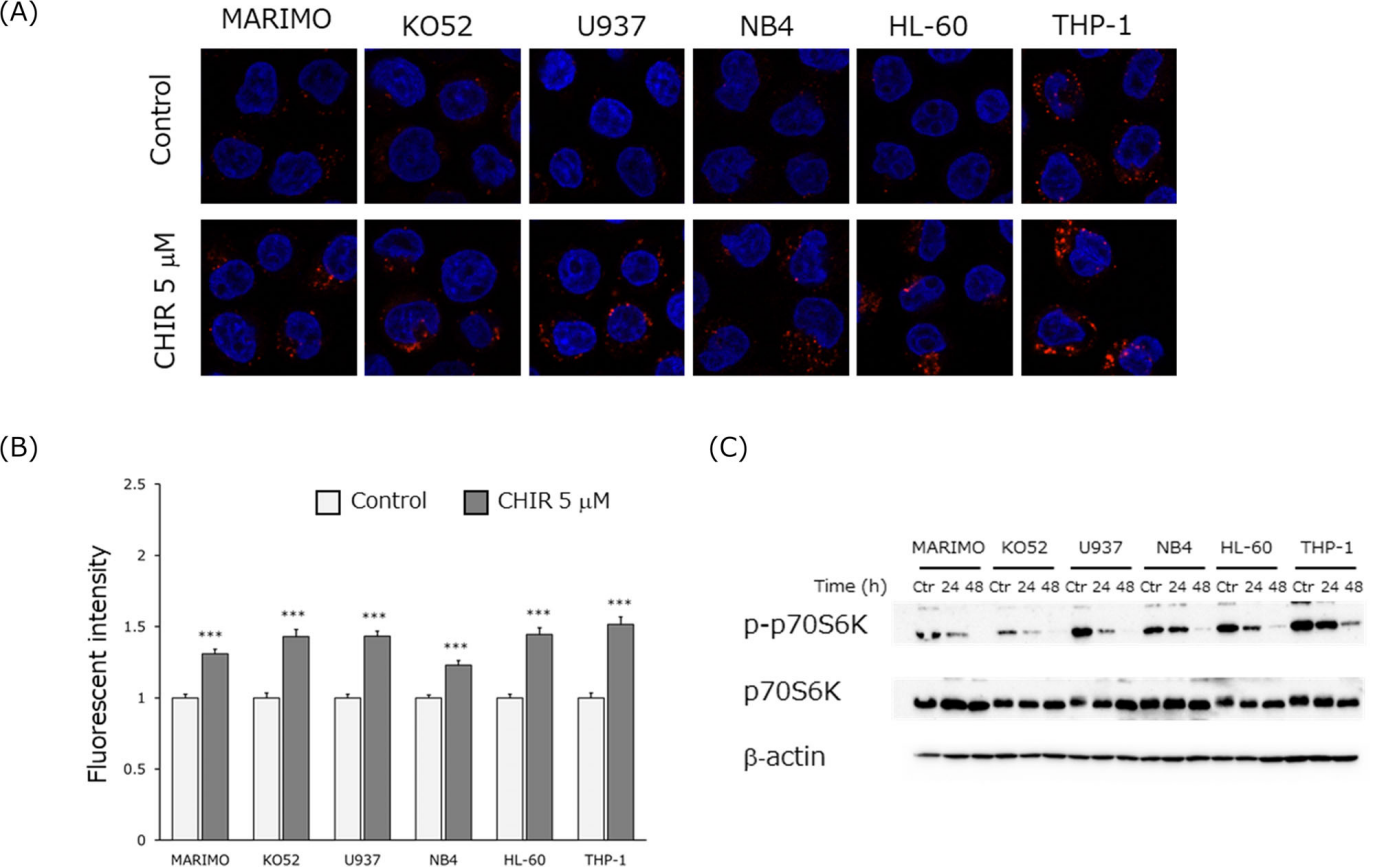
GSK3α/β regulates lysosome biogenesis in an mTORC1‐dependent manner. (A) Cells were exposed to 5 μM CHIR99021 (CHIR) for 24 h, followed by staining with LysoTracker Red DND‐99 (300 nM) for 10 min, fixing with 4% paraformaldehyde, and imaging by confocal microscopy. Untreated cells were used as a control. (B) LysoTracker fluorescent intensity was quantified using ZEN 2 software. Results are shown as the mean ± standard error of the mean (SEM). Statistical analysis was performed by Student's *t*‐test. *, ** and *** indicate *p* < 0.05, *p* < 0.01, and *p* < 0.001, respectively. (C) Cells were treated with 5 μM CHIR for 24 and 48 h. Whole cell lysates were analyzed by western blotting for p‐p70S6K and p70S6K. β‐actin was used as a loading control.

The acidic environment of lysosomes is essential for cleavage of the hydrazone linker in GO, releasing calicheamicin. Lysosomal activity is regulated via mTORC1, but activation of the PI3K/AKT/mTOR pathway in many cancers suggests a decline in lysosomal function. Inhibition of GSK3 activity has been reported to regulate lysosomal activity via mTORC1 [26]. Therefore, we investigated whether CHIR99021 affects lysosomal function within AML cell lines by using LysoTracker Red DND‐99, a dye that accumulates in acidic organelles (lysosomes) and can reveal changes in lysosomal pH. CHIR99021 substantially increased fluorescence intensity (acidity) in all 6 AML cell lines, compared to controls (Figure 3A,B). In MARIMO cells, lysosomal function was not activated by mTORC1/2 inhibitors [19, 20] but was by GSK3α/β inhibitors.

To determine if activation of lysosomal function by GSK3α/β inhibitors is mediated by mTORC1, we monitored phosphorylation of the serine/threonine kinase p70S6K, which is the target of mTORC1. Phosphorylation was suppressed as CHIR99021 in time‐dependent manner in all AML cell lines (Figure 3C), consistent with the notion that GSK3α/β inhibitors enhance the lysosomal function of AML cell lines through suppression of mTORC1 activity.

### 3.4 GSK3α/β inhibition induces apoptosis in GO‐resistant cell lines through suppression of MDR‐1(ABCB1) expression

**FIGURE 4:**
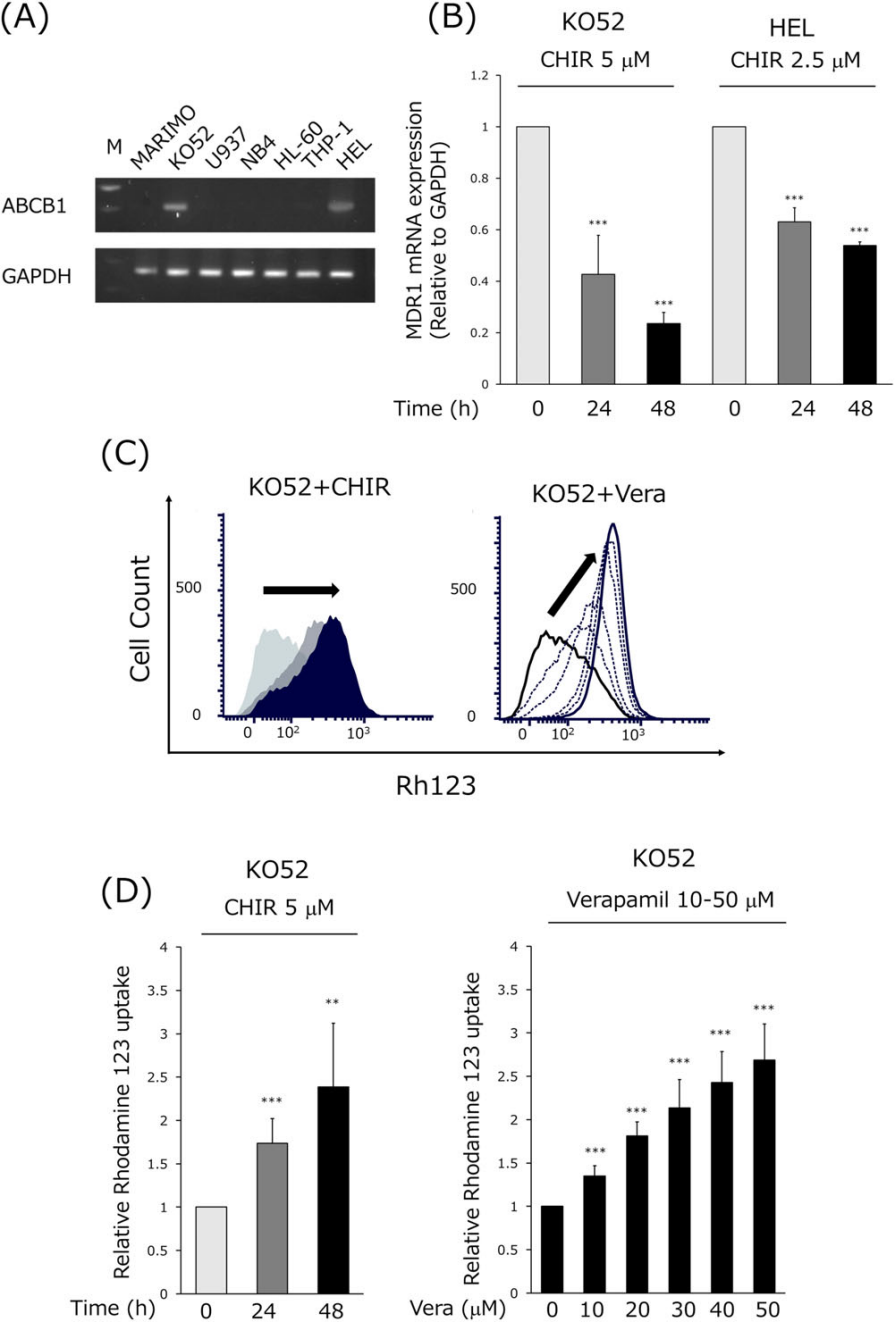
GSK3α/β inhibition induces apoptosis in gemtuzumab ozogamicin (GO)‐resistant cell line through suppression of MDR‐1 expression. (A) Expression of ABCB1 mRNA was analyzed by RT‐PCR. GAPDH was used as the internal control. HEL cells were used as the positive control. (B) KO52 and HEL cells were exposed to CHIR99021 (CHIR) for 24 and 48 h, and then the expression of MDR‐1 mRNA was analyzed by quantitative reverse transcription PCR (qRT‐PCR) and normalized to the expression of GAPDH. Data are expressed as means ± SD of three independent experiments. *** indicates *p* < 0.001. (C) Histograms of flow cytometric analysis of uptake of Rh123 on KO52 cells treated with 5 μM CHIR for 24 and 48 h (left) or treated with 10–50 μM Verapamil (Vera) for 48 h (right). (D) Bar graphs of flow cytometry measuring uptake of Rh123. The heights of the bars are Rh123 MFI from CHIR‐treated cells normalized to Rh123 MFI of untreated cells. Data are expressed as means ± SD of three independent experiments. ** and *** indicate *p* < 0.01 and *p* < 0.001, respectively.

One of the major resistance mechanisms to GO is transport of calicheamicin out of the cell by the multidrug resistance factor MDR‐1(ABCB1) [27, 28]. We first investigated the expression of MDR‐1 (ABCB1) in AML cell lines. Using human erythroleukemia cell line (HEL) cells as controls, we found that KO52 cells expressed MDR‐1 mRNA (Figure 4A). Expression was suppressed after treatment with CHIR99021, both after 24 and 48 h of incubation (Figure 4B). Next, the activity was measured using intracellular Rh123 accumulation as an indicator. The MDR‐1 inhibitor verapamil was used as a control. When KO52 cells were treated with CHIR99021, a time‐dependent increase in intracellular accumulation of Rh123 was observed (Figure 4C,D). When KO52 cells were treated with verapamil and GO in combination, a marked enhancement of the cell‐killing effect of GO was observed. Verapamil had a robust effect in KO52 cells but was less active in MARIMO and U937 cells (in which expression of MDR‐1 could not be confirmed by PCR) (Figure S2). GSK3α/β inhibitors, by suppressing expression of MDR‐1, can inhibit the secretion of calicheamicin and enhance GO cytotoxicity.

### 3.5 GSK3α/β inhibition induces apoptosis in GO‐resistant cell lines through suppression of Bcl‐2 expression

**FIGURE 5:**
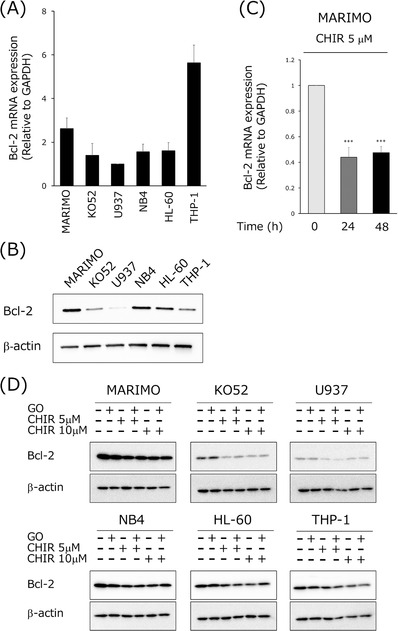
GSK3α/β inhibition induces apoptosis in gemtuzumab ozogamicin (GO)‐resistant cell lines through suppression of Bcl‐2 expression. (A) Bar graph of expression of Bcl‐2 mRNA analyzed by qRT‐PCR and normalized to the expression of GAPDH. The height of each bar is the mean  ±  SD. Each experiment was repeated at least three times. (B) Western blots of whole cell lysates of indicated cell lines analyzed for Bcl‐2. β‐actin was used as a loading control. (C) MARIMO cells were exposed to 5 μM CHIR99021 (CHIR) for 24 and 48 h, and then expression of Bcl‐2 mRNA was analyzed by qRT‐PCR, normalized to the expression of GAPDH. Data are expressed as means ± SD of three independent experiments. *** indicates *p* < 0.001. (D) Cell lines were treated with GO, CHIR, or GO+CHIR for 48 h. Whole cell lysates were analyzed as in (B).

Expression of antiapoptotic factors is thought to be one mechanism of resistance to GO. For this reason, we investigated the expression of Bcl‐2, one of the antiapoptotic factors in AML cells. We found that both Bcl‐2 mRNA and protein levels were high in MARIMO cells (Figure 5A,B). However, when MARIMO cells were treated with CHIR99021, the expression of Bcl‐2 mRNA decreased 24 h after treatment, and this suppression was maintained even after 48 h (Figure 5C). Western blot analysis revealed that CHIR99021 treatment suppressed Bcl‐2 expression at the protein level and that suppression was maintained even with the addition of GO (Figure 5D). We then determined the effect on apoptosis of combining the Bcl‐2 inhibitor venetoclax with GO. Venetoclax substantially enhanced GO‐induced apoptosis in MARIMO cells, which express high levels of Bcl‐2. In KO52 cells, in which Bcl‐2 expression is weaker, Venetoclax alone appeared to enhance apoptosis in a dose‐dependent manner, and, in combination with GO, dose‐dependent enhancement of apoptosis was observed. In U937 cells, venetoclax alone, or in combination with GO, did not enhance apoptosis (Figure S3). GSK3α/β inhibitors thus may enhance cytotoxicity in GO‐resistant cell lines by suppressing the expression of Bcl‐2.

### 3.6 GSK3α/β inhibitors synergize with GO in primary human myeloid leukemia cells

To assess the translational potential of our findings, we investigated the effect of GSK3α/β inhibitors on GO using primary leukemic bone marrow cells from patients with different genetic backgrounds. Primary bone marrow mononuclear cells from 20 AML and 3 MDS patients were harvested. Patient characteristics are summarized in Table 1. Cases 3 and 9 had less than 20% myeloblasts but were diagnosed with AML due to genetic abnormalities specific to AML (*RUNX1‐RUNX1T1* in case 3 and *CBFβ‐MYH11* in case 9). The CD33 antigen was detected in blasts of all patients, although expression levels varied between ∼40‐90%.

**TABLE 1:** Clinical characteristics and combination indices of patients in the study

Case	Age	Sex	disease	NCC (BM) ×103 cells/mm3	Blast %	CD33 %	Genetic abnormality	FLT3/ITD or FLT3/TKD mutation	CI
1	62	M	AML‐MRC	214	26.6	94.0	Single independent clone	–	0.2
2	72	F	AML	34	29.8	86.7	RUNX1‐RUNX1T1	–	0.2
3	47	M	AML	228	17.8	51.6	RUNX1‐RUNX1T1	–	0.3
4	66	M	AML‐MRC	43	60.4	80.0	Single independent clone	–	0.3
5	58	M	AML‐MRC	295	40.8	43.8	Normal	+	0.4
6	72	F	AML‐MRC	33	47.4	54.2	Complex	–	0.4
7	71	M	MDS	160	6.2	92.5	Complex including del (5q)	–	0.5
8	77	M	MDS	180	13.2	84.2	Non‐specific	–	0.5
９	52	M	AML	221	13.2	89.7	CBFb‐MYH11	–	0.5
10	34	M	AML	654	91.4	62.1	Complex	–	0.5
11	76	F	AML	72	23.2	90.2	Complex	–	0.7
12	53	M	AML	140	74.8	84.5	CBFb‐MYH11	–	0.7
13	17	F	AML	703	21.6	62.9	NPM1	–	0.8
14	68	F	AML	176	83.2	69.4	Normal	–	0.9
15	78	M	AML‐MRC	157	82.4	98.3	Single independent clone	–	0.9
16	58	M	MDS	38	11.0	87.0	Complex	–	1.0
17	77	M	AML	278	62.2	99.3	Normal	+	1.1
18	54	M	AML‐MRC	844	49.2	98.4	Normal	+	1.1
19	86	M	AML	162	73.2	91.0	Normal	+	1.2
20	25	M	AML	77	49.8	99.7	Single independent clone	–	1.2
21	69	F	AML	385	78.2	99.3	NMP1	+	1.2
22	48	M	AML‐MRC	183	41.6	99.1	Single independent clone	+	1.3
23	76	F	AML	284	94.4	59.6	CBFb‐MYH11	–	(0.7)

Abbreviations: AML‐MRC, acute myeloid leukemia with myelodysplasia‐related changes; BM, bone marrow; CI, combination index; N/A, not available; NCC, nucleated cell count.

**FIGURE 6:**
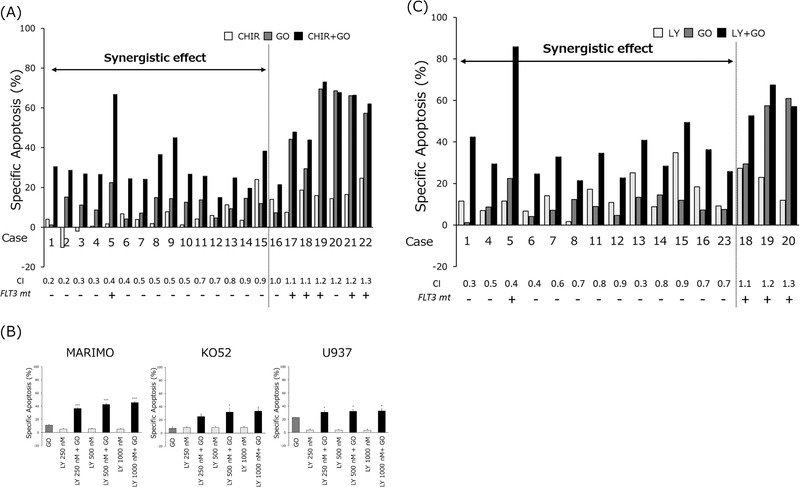
GSK3α/β inhibitors synergize with gemtuzumab ozogamicin (GO) in primary human myeloid leukemia cells. (A) Primary leukemia cells were treated for 48 h with 2.5 μg/ml of GO and 5 or 10 μM of CHIR99021 (CHIR), either separately or in combination, and specific apoptosis was then determined. Numbers below the graph are combination indices (CIs). The presence or absence of *FLT3* mutation (*FLT3 mt*) for each patient is shown as + or −, respectively. CI values were interpreted as additive (CI = 1), synergistic (CI < 1) or antagonistic (CI > 1). (B) MARIMO, KO52, and U937 cells were treated for 48 h with GO, LY2090314 (LY), or GO+LY, and then the levels of specific apoptosis were determined. Data are expressed as means ± SD of three independent experiments. The statistical significance of differences observed between GO and GO+LY was determined using Student's *t*‐test. * and *** indicate *p* < 0.05 and *p* < 0.001, respectively. (C) Primary leukemia cells were treated for 48 h with 2.5 μg/ml of GO and 500 or 1000 nM of LY, either separately or in combination, and the levels of specific apoptosis then were determined. Numbers and symbols below the graph, as well as CI value interpretation, were as in (A).

Treatment of these primary AML cells with GO (2.5 μg/ml) or CHIR99021 (5 or10 μM), singly and in combination, resulted in synergistic effects in 15 of 22 cases, additive effects in 1 case, and antagonistic effects in 6 cases (Figure 6A). Of the six cases with antagonistic effects, five had *FLT3‐ITD* or *FLT3‐TDK* mutations.

We next tested LY2090314, a GSK3 inhibitor used clinically. The effect of GO on cell lines was investigated at concentrations not exceeding the C_max_ used in clinical trials. Similar to previous GSK3 inhibitors, GO cytotoxicity was enhanced in MARIMO, KO52, and U937 cells (Figure 6B). We then investigated the effects of GO and LY2090314 in combination using patient AML cells. Primary AML cells from 16 patients were treated with GO 2.5 μg/ml and LY2090314 at 500 or 1000 nM. In 13 of 16 cases, a synergistic effect was observed. We note that *FLT3* mutations were found in all three cases with antagonism (Figure 6C).

GSK3 inhibitors thus act synergistically with GO to enhance tumor cell killing in AML cells derived from various patients. This synergism was observed in ∼90% of patients, especially in the absence of *FLT3* mutations.

## 4 DISCUSSION

We sought to determine if GO treatment for AML could be augmented through complementation with cytotoxicity enhancers. Previous work had suggested that drugs targeting PI3K/AKT/mTOR signaling pathways might augment the effect of GO, and indeed, we identified drugs that did precisely that. These included CHIR99021, LY2090314, ADZ2858 and AZD1080. In studies using six different AML cell lines, we found that the GSK3α/β inhibitor CHIR99021 provided the most effective enhancement of GO cytotoxicity.

We began by determining the effects on apoptosis of GO alone or in combination with the GSK3α/β inhibitor CHIR99021. In all cell lines used, CHIR99021 at low concentrations synergistically enhanced cell killing by GO. Other GSK3 inhibitors AZD2858, AZD1080 and LY2090314 also augmented cytotoxicity. We note that our previous attempts to enhance GO toxicity using the mTORC1/2 inhibitor, which activates lysosomal function, failed in MARIMO and KO52 cells [19, 20]. Because the expression of CD33 in MARIMO is the lowest among our cell lines, it was possible that suppression of GO internalization underlay GO resistance. We also hypothesized that GO resistance in KO52 cells might be due to the extracellular excretion of calicheamicin, the payload of GO, due to the expression of MDR‐1. Both of these effects were mitigated by GSK3 inhibitors.

Previous studies have shown a correlation between CD33 expression and GO efficacy in vitro [24, 29]. In contrast, inconsistent results have been seen in vivo [10, 15, 28, 30]. This may be due to differences in the criteria used to define CD33 positivity [25]. Our results show that GSK3 inhibition induces CD33 expression in four of six cell lines. In particular, in MARIMO cells, almost no cell‐killing effect was observed with GO alone, whereas GSK3 inhibitors significantly increased CD33 expression and thereby enhanced cell killing by GO. This presumably was due to the induction of CD33 expression by the GSK3 inhibitor, which supports intracellular uptake of GO. However, the mechanism by which GSK3 inhibitors are involved in CD33 expression is unknown.

Because GSK3α/β activity has been shown to affect lysosomal function [31, 32, 33, 34, 35], which is critical for scission of the link between GO and its toxic partner, calicheamicin, we asked whether activation of the PI3K/AKT/mTOR pathway would enhance the effects of GO by decreasing intra‐organellar pH. A positive effect was observed in all 6 cell lines. A fundamental factor in lysosome function is the level of lysosome biosynthesis. mTORC1 suppresses lysosomal biosynthesis by phosphorylation of the transcription factor EB (TFEB) [35, 36]. On the other hand, the activity of mTORC1 is suppressed through Rheb suppression by the TSC1‐TSC2 complex [31, 37]. Since TSC2 is also a target for GSK3, inhibition of GSK3 activity also causes mTORC1 inactivation [34]. In AML cell lines, CHIR99021 reduces phosphorylation of p70S6K, suggesting that mTORC1 is inactivated and lysosomal function is activated through the above pathways. In addition, GSK3 can directly phosphorylate TFEB [36]. Therefore, GSK3 inhibitors can also activate lysosomal function via dephosphorylation of TFEB, independent of the mTORC1 pathway. We have previously reported that MARIMO's lysosomal function was not activated by mTORC1/2 inhibitors [19, 20]. However, results reported here show that GSK3 inhibitors are lysosomal activators. In MARIMO (for unknown reasons), activation of lysosomal function may be induced mainly through direct TFEB dephosphorylation by GSK3 inhibition rather than mTORC1 inhibition.

It is known that the ABC (ATP‐binding cassette) transporter is involved in drug excretion. It also has been reported that MDR‐1, and to a lesser extent, MRP1, are key factors involved in the excretion of calicheamicin [38, 39]. Expression of MDR‐1 is regulated via T cell factor/lymphoid enhancer factor by nuclear‐translocated β‐catenin [40, 41]. Since GSK3 inhibition increases β‐catenin in the nucleus, one would expect the expression level of MDR‐1 to increase with GSK3 inhibition. It has been reported in chronic myelogenous leukemia that GSK3 inhibition does increase the amount of ABCB1 (MDR‐1) mRNA through activation of the WNT/ β‐catenin pathway [42]. We, however, see the opposite result. The explanation for this remains unclear.

One mechanism of GO resistance is the expression of antiapoptotic factors such as Bcl‐2. We confirmed that CHIR99021 suppresses the expression of Bcl‐2 at the mRNA and protein levels. In MARIMO cells, in which Bcl‐2 expression is high, CHIR99021, in combination with GO, decreased Bcl‐2 expression, overcoming GO resistance. GSK3β has been reported to promote nuclear localization of NFκB and regulate the expression of antiapoptotic factors in some cancers, including AML [43, 44, 45]. We observed that GSK3β inhibitors suppressed Bcl‐2 expression, but caused strong cytotoxicity, which may explain why no significant enhancement was obtained when GSK3β inhibitors were used in combination with GO (data not shown).

Our most exciting results came from testing the effects of GSK3α/β inhibitors and GO on bone marrow mononuclear cells taken directly from AML patients. We observed a strong synergistic effect in 68% (CHIR99021) or 81% (LY2090314) patients. This effect was observed irrespective of the type of gene mutation. In addition, it has also been seen in cases with complex karyotypes that predict poor prognosis, suggesting that the combination of GO and GSK3 inhibitors is effective in treating AML. On the other hand, no synergistic effect was observed in the case of FLT3 mutations. This may be due to the originally high expression levels of CD33 and lysosomal activity in these cases [20, 46]. Sufficient cell death may have been induced by GO alone, which may have obscured the effect of GSK3 inhibitor.

CHIR99021 is currently an experimental reagent, whereas LY2090314 is a drug in clinical trial use. In Phase 2 clinical trials, LY2090314 showed acceptable safety in patients with AML, but has not been approved as monotherapy due to limited clinical benefit. This suggests that combining GO with CHIR99021 or other GSK3 inhibitors should be beneficial and that we can expect the development of combination therapies with GO [47].

We have found that GSK3 inhibitors overcome GO resistance through multiple means, including increasing CD33 expression, stimulating lysosomal function, decreasing MDR‐1 expression, and suppressing Bcl‐2 expression. However, the precise mechanisms of these actions remain to be elucidated. Variations in the GO enhancing effect among various GSK3 inhibitors may be due to the balance between the inhibitory effects of GSK3α and GSK3β. GSK3β inhibition alone may not be sufficient to augment the cytotoxicity of GO. To optimize GO activity, it will be necessary to clarify the roles of GSK3α and GSK3β in GO resistance and to develop new GSK3α/β inhibitors based on these results.

In combination with a GSK inhibitor, GO showed similar cytotoxicity to U937 cells at as low as one‐fifth the concentration for GO when used as a single agent (data not shown). Thus, it may be possible to use lower doses of GO, in combination with GSK3 inhibitors, to treat older patients who suffer from severe toxicity at standard GO doses. It is also currently used in combination with daunorubicin and cytarabine, but replacing these with a less toxic GSK3 inhibitor may produce similar or stronger effects while reducing side effects.

In addition, resistance, which is a major problem in GO treatment, may be overcome by using it in combination with a GSK3 inhibitor. Among attempts to overcome ADC resistance, improvements in ADC linkers and payloads have been the most commonly used, and have shown the greatest success [18, 48]. Further, attempts are being made to overcome the ADC resistance mechanism itself with other drugs. For example, MDR‐1 inhibitors can be expected to increase the intracellular concentration of payload [49]. Also, HSP90 inhibitors are known to block endosomal recycling and are expected to increase trafficking of Her2 and antibody complexes [50]. Accordingly, combinations of several ADCs with MDR‐1 inhibitors or HSP90 inhibitors are expected to show clinical efficacy. Of concern, however, some agents, such as MDR‐1 inhibitors and Bcl‐2 inhibitors, can counter only one mode of resistance, limiting their effectiveness. However, as our present findings show, combination with GSK3 inhibitors would increase the range of patients who could benefit. Combination therapy may also be effective with other ADCs having lysosomally degradable linkers and similar mechanisms of resistance against the drug payload.

## AUTHOR CONTRIBUTIONS

AI designed and performed experiments, analyzed and interpreted the data, and wrote the manuscript. YM, SK, HG, KK, SK, KY, HM, and HM provided critical feedback and support. YM, SK, YM, YM, SO, HM, AK, RS, KK, AH, SN, HG, KK, SK, and KY collected patient samples and data. HM supervised the project. All authors reviewed the manuscript and approved its final version.

## CONFLICT OF INTEREST

HM has a COI with Pfizer (honoraria). All other authors declare no conflict of interest with the work presented in this study.

## Supporting information

SUPPORTING INFORMATION

## Data Availability

The data that support the findings of this study are available from the corresponding author upon reasonable request.
