# Supplementary material for: GSK3 inhibitor enhances gemtuzumab ozogamicin‐induced apoptosis in primary human leukemia cells by overcoming multiple mechanisms of resistance
Source: EJHaem. 2022 Dec 12;4(1):153–64. doi: 10.1002/jha2.600 (PMC9928658; doi:10.1002/jha2.600)
Supplement: Supplementary file 1 — SUPPORTING INFORMATION [file JHA2-4-153-s001.docx]

Supplementary information for manuscript:

**GSK3 inhibitor enhances gemtuzumab ozogamicin-induced apoptosis in primary human leukemia cells by overcoming multiple mechanisms of resistance**

**This supplementary information includes:**

1. Supplementary Table S1
2. Supplementary Table S2
3. Supplementary Methods
4. Supplementary Figure Legends
5. Supplementary Figures S1 to S3
6. References
7. **Supplementary Table S1:** Chemicals and antibodies

|  | Name | Cat. |
| --- | --- | --- |
| Reagents | Gemtuzumab ozogamicin | Pfizer Inc. |
|  | CHIR99021 | Focus Biomolecules 252917-06-9 |
|  | AZD1080 | AOBIOUS, INC. 612487-72-6 |
|  | AZD2858 | BLD Pharm. 486424-20-8 |
|  | LY2090314 | Cayman Chem. 603288-22-8 |
|  | Tideglusib | Sigma-Aldrich 865854-05-3 |
|  | BRD0705 | MCE 2056261-41-5 |
|  | Verapamil | Sigma-Aldrich 152-11-4 |
|  | Rhodamine 123 | Sigma-Aldrich 62669-70-9 |
|  | Venetoclax | Abcam ab217298 |
|  | SCF | PeproTech #300-07 |
|  | IL-3 | PeproTech #200-03 |
|  | IL-6 | PeproTech #200-06 |
|  | TPO | PeproTech #300-18 |
|  | FLT3l | PeproTech #300-19 |
| Antibodies | phospho-p70S6K(Thr389) | #9205, Cell Signaling |
|  | p70S6K | #9202, Cell Signaling |
|  | β-actin | A1978, Sigma-Aldrich |
|  | BCL-2 | #2872, Cell Signaling |
|  | PARP | #9542, Cell Signaling |
|  | CD33 Monoclonal Antibody (P67.6), FITC | #11-0337-42, eBioscience |
|  | Mouse IgG1 kappa Isotype Control FITC | #11-4714-42, eBioscience |

1. **Supplementary Table S2:** Primers used for real time PCR analysis.

|  | Forward | Reverse |
| --- | --- | --- |
| ABCB1 | CCCATCATTGCAATAGCAGG | GTTCAAACTTCTGCTCCTGA |
| GAPDH | GTCTCCTCTGACTTCAACAGCG | ACCACCCTGTTGCTGTAGCCAA |
| Bcl-2 | ATCGCCCTGTGGATGACTGAGT | GCCAGGAGAAATCAAACAGAGGC |
| β-actin | CACCATTGGCAATGAGCGGTTC | AGGTCTTTGCGGATGTCCACGT |

1. **Supplementary Methods**

**Confocal microscopy**

Cells were seeded at a concentration of 2.5 × 10^5^ cells/mL and exposed to 5 μM CHIR99021 for 24 hours. The treated cells were fixed and imaged as reported previously (1, 2).

**RNA extraction and RT-PCR**

AML cell lines were seeded in growth medium at a density of 2.5 × 10^5^ cells/mL and harvested 48 hours later. MARIMO and KO52 cells each were exposed to CHIR99021 5 µM for 24 or 48 hours and then harvested. Total RNA was extracted from these cells using NucleoSpin® RNA kit (Macherey-Nage, Dueren, Germany) in accordance with the manufacturer's protocol. cDNA was synthesized from the extracted RNA using the PrimeScript ™ RT reagent Kit (Perfect Real Time) (Takara Bio Inc., Shiga, Japan). PCR was performed using the ABCB1 primer with the synthesized cDNA. GAPDH was used as a control.

**Western Blotting**

To assess the phosphorylation of p70S6K, AML cell lines were seeded in growth medium at a concentration of 2.5 × 10^5^ cells/mL and exposed to CHIR99021 5 µM for 24 or 48 hours. Cells were exposed to GO and CHIR99021 for 48 hours, individually or in combination, at concentrations of 5 µM or 10 µM to assess PARP cleavage and Bcl-2 expression. The collected cells were treated and analyzed as reported previously (1, 2).

1. **Supplementary Legends**

**Supplementary Figure S1:** **GSK3α/β inhibitor enhances the cytotoxicity effect of GO in different leukemia cell lines.**

(a) Combination index (CI) values of GO and CHIR99021 (CHIR) at the indicated concentrations. CI values were interpreted as additive (CI = 1), synergistic (CI < 1) or antagonistic (CI > 1). (b) U937, MARIMO and KO52 cells were treated with AZD2858 (A2858) for 48h, and then specific apoptosis was determined. Data are expressed as means ±SD of three independent experiments. (c) U937, MARIMO and KO52 cells were treated with AZD1080 (A1080) for 48h, and then specific apoptosis was determined. Data are expressed as means ±SD of three independent experiments.

**Supplementary Figure S2:** **GSK3α/β inhibition induces apoptosis in GO-resistant cell line through suppression of MDR-1 expression.**

(a) MARIMO, KO52, and U937 cells were treated with GO, Verapamil (Vera), or GO+Vera for 48h, and then specific apoptosis was determined. Data are expressed as means ±SD of three independent experiments. The statistical significance of differences observed between GO and GO+Vera was determined using Student's t-test. *, ** and *** indicate p<0.05, p<0.01 and p<0.001, respectively.

**Supplementary Figure S3:** **GSK3α/β inhibition induces apoptosis in GO-resistant cell lines through suppression of Bcl-2 expression.**

(a) MARIMO, KO52, and U937 cells were treated with GO, Venetoclax (Ven), or GO+Ven for 48h, and then specific apoptosis was determined. Data are expressed as means ±SD of three independent experiments. The statistical significance of differences observed between GO and GO+Ven was determined using Student's t-test. *, **, and *** indicate p<0.05, p<0.01 and p<0.001, respectively.

1. **Supplementary Figures S1 to S3**

**
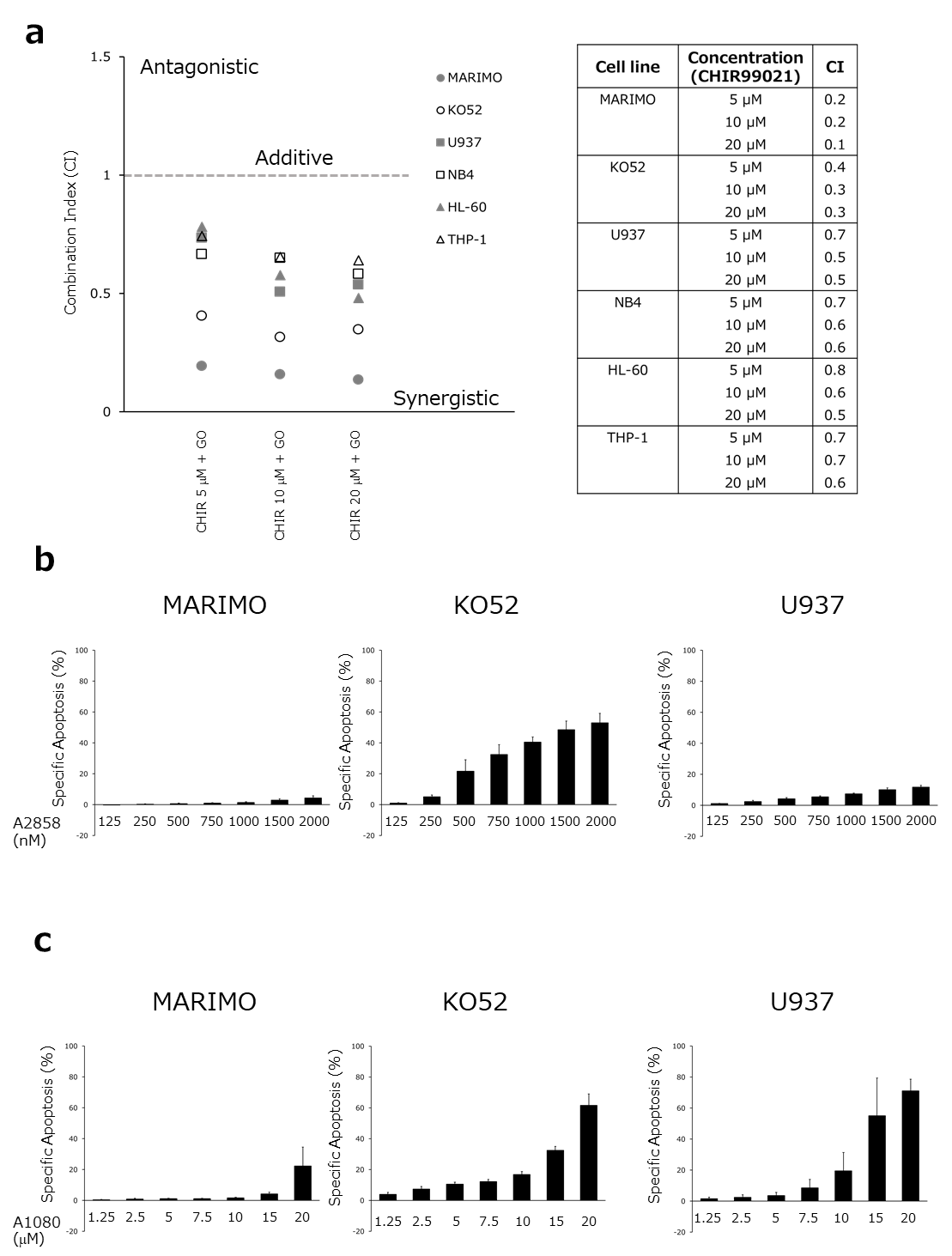
**

**Figure S1**

**
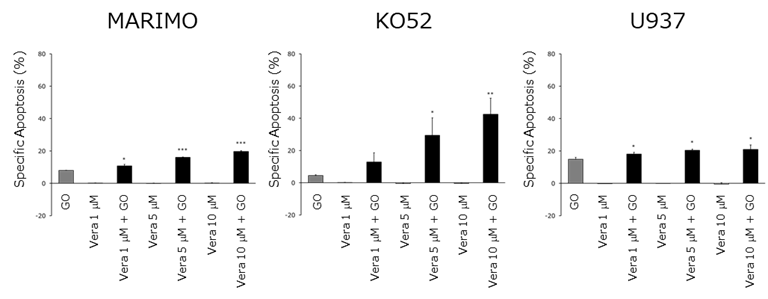
**

**Figure S2**

**
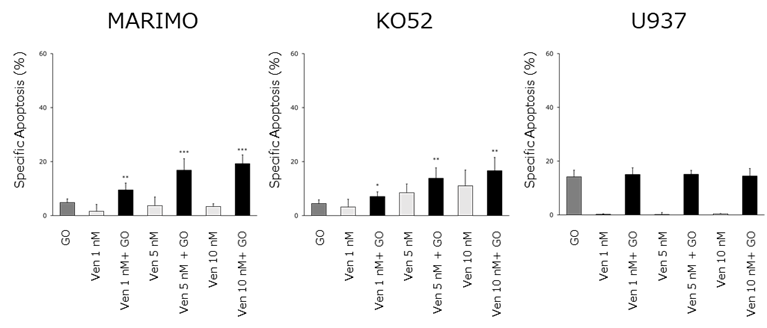
**

**Figure S3**

1. **References**
2. Mizutani Y, Inase A, Maimaitili Y, Miyata Y, Kitao A, Matsumoto H, et al. An mTORC1/2 dual inhibitor, AZD2014, acts as a lysosomal function activator and enhances gemtuzumab ozogamicin-induced apoptosis in primary human leukemia cells. Int J Hematol. 2019;110(4):490-9.
3. Klco JM, Spencer DH, Lamprecht TL, Sarkaria SM, Wylie T, Magrini V, et al. Genomic impact of transient low-dose decitabine treatment on primary AML cells. Blood. 2013;121(9):1633-43.
